# Cervical Necrotizing Fasciitis With Intractable Hiccups

**DOI:** 10.7759/cureus.88145

**Published:** 2025-07-17

**Authors:** Eduardo Lorenzo, Parikshit Chapagain, Swotantra Gautam, Alexander Herbert

**Affiliations:** 1 Internal Medicine, AdventHealth Orlando, Orlando, USA; 2 General Medicine, B.P. Koirala Institute of Health Sciences, Dharan, NPL

**Keywords:** cervical necrotizing fasciitis, immunocompromised, intractable hiccups, misdiagnosis, neck mass, necrotizing fasciitis, rheumatoid arthritis, sepsis

## Abstract

We report a case of a 61-year-old immunosuppressed man with rheumatoid arthritis on etanercept and past intravenous drug use who presented with intractable hiccups, an unreported feature of cervical necrotizing fasciitis (CNF). He was initially diagnosed with a viral syndrome, but later developed severe sepsis with neck swelling, dysphagia, and ligneous induration. The absence of skin changes, probably secondary to etanercept-induced immunosuppression, hindered diagnosis. Contrast-enhanced computed tomography (CT) scan of the neck indicated CNF accompanied by descending necrotizing mediastinitis (DNM) with gas in the cervical and mediastinal soft tissues. The early surgical debridement, empirical broad-spectrum antibiotics (linezolid, ampicillin-sulbactam), and multidisciplinary teamwork were responsible for a good outcome after delayed diagnosis. Hiccups that can be regarded as a soft sign of phrenic/vagus nerve irritation or hyponatremia caused by sepsis were introduced as another aspect of this disease. This case illustrates the need for a high index of clinical suspicion and the utility of early imaging in immunocompromised patients with atypical presentations. These findings indicate a need for new diagnostic strategies to enhance the outcome of this deadly disease.

## Introduction

Cervical necrotizing fasciitis (CNF) is a rare and aggressive soft-tissue infection of the head and neck, accounting for approximately 2.6% of all soft-tissue infections, with a mortality rate ranging from 7% to 20% [1,2]. When complicated by descending necrotizing mediastinitis (DNM), the prognosis worsens significantly due to rapid spread to the mediastinum, often leading to septic shock [1,2]. Predisposing factors include diabetes mellitus, immunosuppressive therapy (e.g., etanercept), renal failure, malignancy, older age, odontogenic infections, intravenous drug use (IVDU), pharyngeal infections, and trauma [3,4].

Clinically, CNF typically presents with severe neck pain, swelling, erythema, edema, and pain disproportionate to physical findings, followed by systemic symptoms such as fever, malaise, and toxicity [4]. Physical examination may reveal cutaneous inflammation, induration with a “sclerous” consistency, skin discoloration, bullae, cutaneous gangrene, or crepitation in cases involving gas-forming organisms [5]. These features distinguish CNF from cellulitis, as noted by the Infectious Diseases Society of America [5]. However, atypical presentations are common, particularly in immunocompromised patients, where attenuated inflammatory responses may obscure classic signs [2,6].

Risk factors such as immunosuppression and IVDU are well-documented, with odontogenic sources frequently implicated in polymicrobial infections involving *Streptococcus* spp., anaerobes, and *Actinomyces* [1,7]. Despite extensive literature, intractable hiccups have not been reported as a presenting symptom of CNF, making this case novel. We describe a case of CNF with DNM in an immunocompromised patient presenting with hiccups, highlighting diagnostic challenges, the necessity for prompt treatment, and the value of a multidisciplinary approach.

## Case presentation

A 61-year-old male with a 10-year history of rheumatoid arthritis (RA), treated with etanercept for seven years, presented with features suggestive of a severe infection. His social history included a 30-pack-year smoking history, daily alcohol consumption (six to 12 beers), and prior IVDU, which, combined with RA-related immunosuppression, increased his risk of serious infections. He had a history of appendectomy and recurrent herpes labialis.

The patient initially experienced non-specific symptoms for approximately two weeks, including intractable hiccups, sudden-onset vertigo, chills, and vomiting, with a recorded temperature of 102°F (38.9°C). These symptoms, while persistent, could initially be interpreted as a prolonged viral syndrome, a common clinical presentation. This led to an initial misdiagnosis of a viral syndrome at an outside facility, where imaging and laboratory tests were not performed, and he was discharged with supportive care advice, delaying the diagnosis of his underlying condition.

Within days, his condition deteriorated, with neck swelling, dysphagia, a muffled voice, progressive severe pain, and signs of sepsis necessitating urgent admission. On examination, he was toxic, poorly responsive, and exhibited dysphonia. His neck was markedly swollen with a deviated uvula to the left and palpable “wooden-hard” induration, consistent with CNF. Notably, cutaneous signs such as erythema, bullae, or gangrene were absent, complicating early recognition.

**Figure 1:**
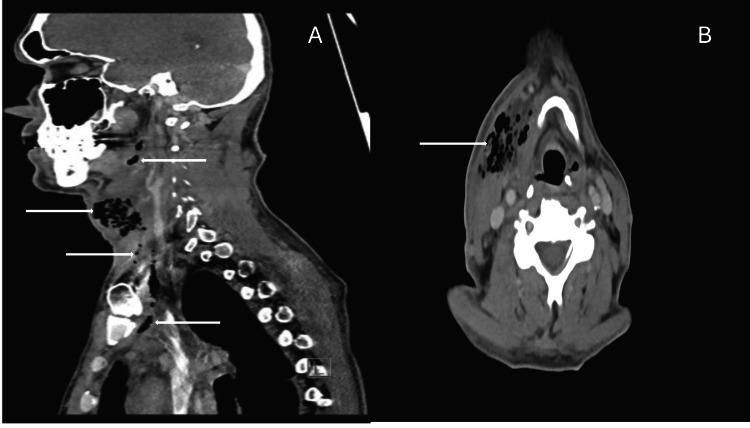
Initial CT scan views demonstrating cervical necrotizing fasciitis and descending necrotizing mediastinitis. (A) Sagittal contrast-enhanced CT scan view shows extensive gas formation (white arrows) throughout the right deep neck spaces, including the parapharyngeal, submandibular, and visceral spaces, with trace extension into the superior mediastinum. (B) Corresponding axial CT scan view from the same study further demonstrates multiple foci of gas (white arrow) within the deep tissues of the right neck, highly suspicious for rapidly progressing necrotizing fasciitis.

Initial laboratory studies on admission demonstrated a white blood cell count of 26.6 x 10³/μL (reference range: 4.0-11.0) with 91.4% neutrophils (reference range: 40.0-75.0), which reflected severe inflammation. The serum sodium was 128 mmol/L (reference range: 135-145); sepsis-induced syndrome of inappropriate antidiuretic hormone secretion (SIADH). The C-reactive protein was also raised at 423.40 mg/L (reference value: <10.0), indicating a large amount of tissue damage. Broad-spectrum antibiotics were initiated without delay. A contrast-enhanced CT scan of the neck (Figure 1), conducted on admission, demonstrated multiple foci of gas, mainly within the right deep neck spaces, including the parotid, parapharyngeal, submandibular, and visceral spaces. Gas was observed extending downwards into the right supraclavicular space and minimal extension into the superior mediastinum (Figure 1). The findings were highly indicative of CNF and DNM.

**Figure 2:**
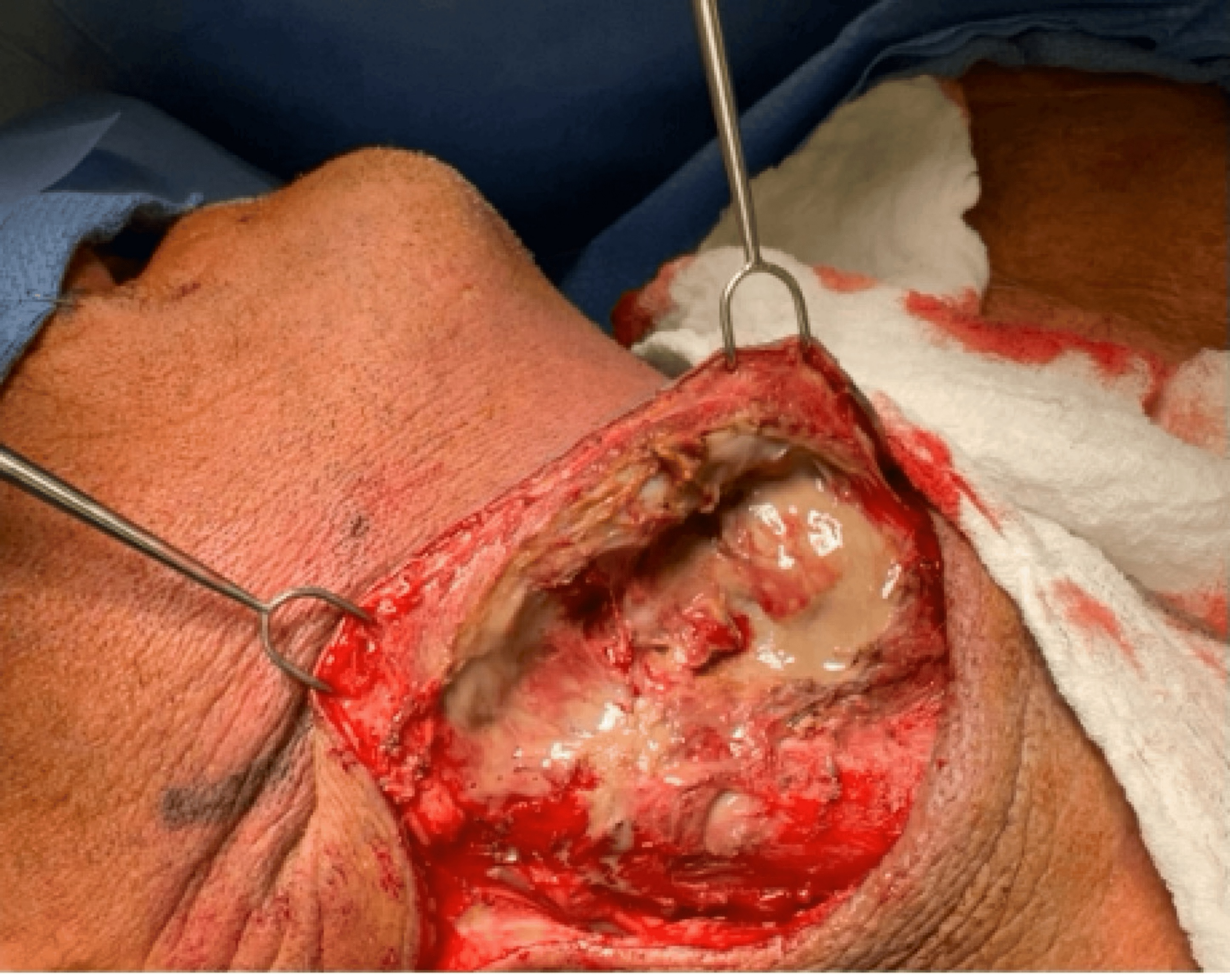
Intraoperative findings during emergent neck and mediastinal exploration. Intraoperative view during neck exploration demonstrating copious purulence and extensive necrosis of cervical and superior mediastinal tissues, consistent with necrotizing fasciitis.

Emergent surgical treatment was initiated on the same day of admission, involving right neck exploration, drainage of the parapharyngeal abscess, superior mediastinal exploration, limited right neck dissection of levels 2, 3, and 4, and deep neck debridement (Figure 2). Intraoperative findings revealed copious purulence and widespread necrosis of the cervical and superior mediastinal soft tissues, consistent with necrotizing fasciitis. Despite the severely infected process, vital neurovascular structures were preserved, and there was no postoperative neurological deficit. Drains were inserted into the parapharyngeal space and superior mediastinum.

**Figure 3:**
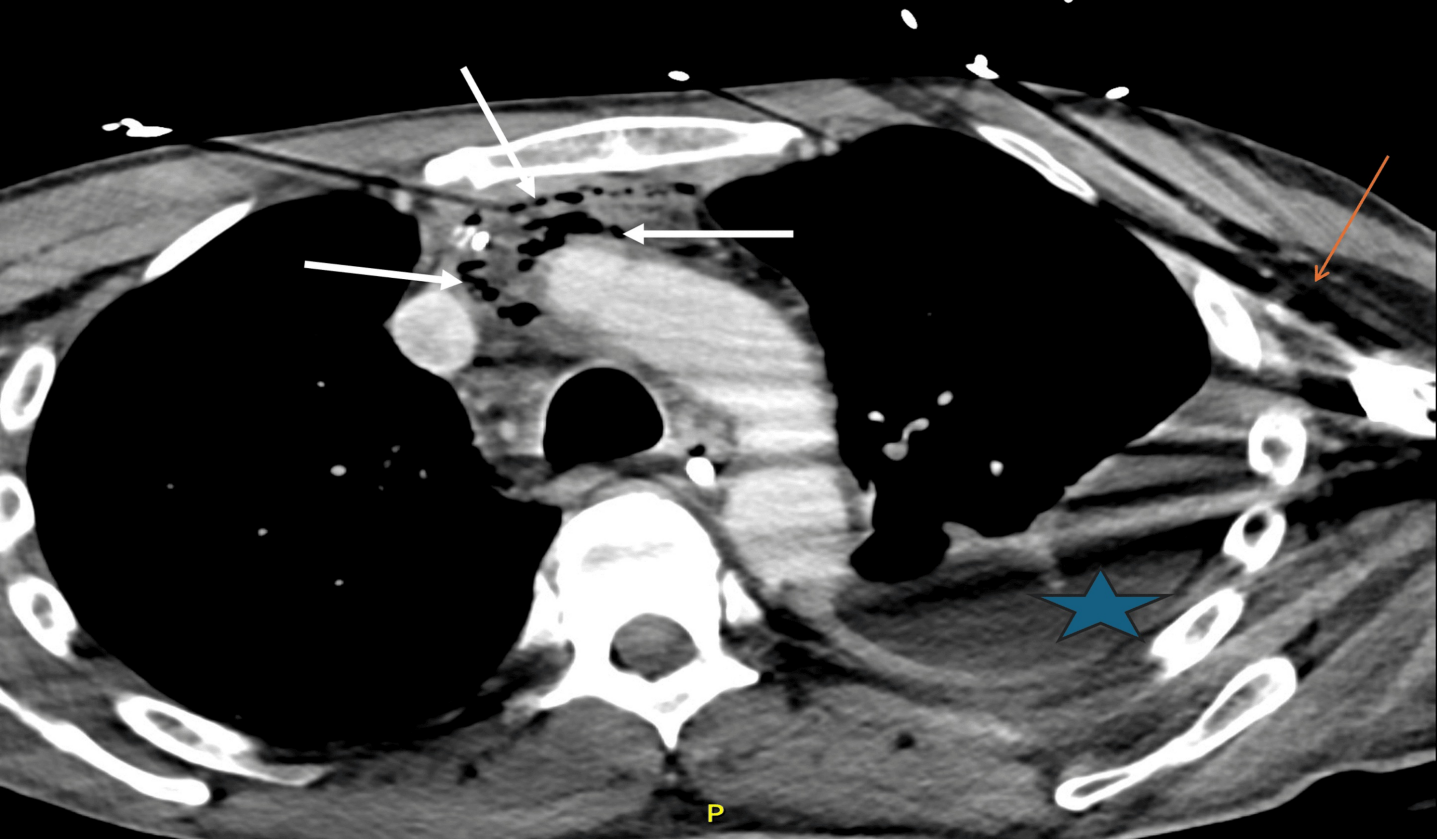
Follow-up axial CT scan demonstrating progressive necrotizing fasciitis and mediastinal involvement. Axial contrast-enhanced CT scan (two days post-initial surgery) demonstrating interval progression of necrotizing fasciitis. This view shows extensive gas foci (white arrows) within the anterior mediastinum and right upper chest, with new gas foci (red-orange arrow) in the left upper chest and axilla, along with a left pleural effusion (blue star). These findings indicate a persistent and spreading infection despite initial debridement.

The following day, broad-spectrum antibiotics were tailored to linezolid and ampicillin-sulbactam, guided by culture results that identified *Streptococcus constellatus*, *Parvimonas*, *Cutibacterium*, and *Actinomyces turicensis*, consistent with an odontogenic source. Following initial surgical debridement and antibiotic therapy, the patient's white blood cell count improved, decreasing to 8.4 x 10³/μL. His C-reactive protein also dropped to 115.40 mg/L, and his hyponatremia was corrected with aggressive fluid resuscitation. However, a follow-up CT scan performed just two days later (Figure 3) revealed a concerning interval progression of the infectious process. It showed persistent, multiple foci of gas within the right neck, notably extending into the right floor of the mouth, along with newly developed gas foci along the left side of the neck. Substantial gas was again noted within the mediastinum, accompanied by progressive fat stranding and thickening of fascial planes. These findings, indicating residual and spreading infection, directly guided further surgical management.

The patient had a prolonged hospital stay but was eventually discharged in stable condition with comprehensive home care support, on ampicillin-sulbactam for persistent infection and metoprolol for coronary risk reduction. He was referred for follow-up with infectious disease, oral/maxillofacial surgery, and thoracic surgery to monitor for recurrence or complications.

## Discussion

CNF with DNM is a rare, life-threatening infection with mortality rates of 7-20% [1,2]. This case of a 61-year-old immunocompromised male with RA on etanercept, a history of IVDU, and intractable hiccups as the primary presenting symptom highlights the diagnostic challenges of atypical CNF presentations. The initial misdiagnosis as a viral syndrome, due to nonspecific symptoms like hiccups, vertigo, chills, and vomiting, led to a critical delay in recognizing severe sepsis, emphasizing the need for a high index of suspicion in immunocompromised patients.

Etanercept, a tumor necrosis factor-alpha (TNF-α) inhibitor, suppresses key inflammatory responses, masking classic CNF signs such as erythema, bullae, or crepitation, which are present in 70-80% of cases [4,5]. This immunosuppression, combined with IVDU and chronic alcohol use, likely accelerated the infection’s progression by impairing immune defenses and increasing susceptibility to polymicrobial infections [6]. Beyond TNF-α inhibitors, other immunosuppressive agents (e.g., corticosteroids, calcineurin inhibitors, and antimetabolites) or conditions like severe neutropenia, advanced HIV, or uncontrolled diabetes can similarly obscure typical inflammatory responses and delay diagnosis of severe infections. The polymicrobial culture (*Streptococcus constellatus*, *Parvimonas*, *Cutibacterium*, and *Actinomyces turicensis*) suggests an odontogenic source, consistent with 30-50% of CNF cases [7,8], though direct inoculation via IVDU remains a possible alternative, as contaminated needles can introduce oral and skin flora [6].

The absence of cutaneous manifestations early in the disease course delayed clinical suspicion, with “wooden-hard” induration and neck swelling emerging later as critical clues. This underscores the importance of considering CNF in immunocompromised patients with nonspecific symptoms and risk factors like odontogenic infections or IVDU.

Intractable hiccups, a novel presenting symptom in CNF, may have several explanations. Phrenic nerve (C3-C5) irritation from the parapharyngeal abscess or cervical necrosis is a likely mechanism, as inflammation or compression near the nerve’s course can trigger diaphragmatic spasms [9]. Alternatively, DNM may have irritated mediastinal structures, including the vagus nerve, which has afferent pathways linked to the hiccup reflex arc [10]. Sepsis-induced hyponatremia (128 mmol/L) could also contribute, as electrolyte imbalances disrupt neuromuscular function and may provoke hiccups [11]. Central nervous system involvement, though less likely given the absence of neurological deficits, could involve the brainstem’s hiccup reflex center in severe infections [10]. The resolution of hiccups post-debridement suggests a direct link to the infectious process, with phrenic or vagus nerve irritation being the most plausible mechanisms given the anatomical involvement.

The Laboratory Risk Indicator for Necrotizing Fasciitis (LRINEC) score, retrospectively calculated as 10, indicated a high probability of necrotizing fasciitis (>75%) based on elevated white blood cell count (26.6 x 10³/μL), C-reactive protein (423.40 mg/L), and hyponatremia [3]. Early use of the LRINEC score could have prompted timely imaging and specialist referral, potentially averting progression to severe sepsis. With a sensitivity of 80-90% and specificity of 75-85%, the LRINEC score is a valuable triage tool, but its reliance on immediate laboratory access limits its utility in urgent care settings [3].

This case highlights the need for routine LRINEC scoring in patients with suspected soft tissue infections and risk factors, alongside comprehensive history-taking to identify odontogenic or injection-related triggers. The successful outcome, despite diagnostic delay, underscores the efficacy of aggressive multimodal therapy. Contrast-enhanced CT imaging was pivotal, revealing gas in the cervical and mediastinal tissues, confirming CNF with DNM [2].

Emergent surgical debridement, including cervicotomy and mediastinal exploration, achieved source control, preserving neurovascular structures. Broad-spectrum antibiotics (linezolid and ampicillin-sulbactam), tailored to culture results, targeted the polymicrobial infection [12]. Aggressive fluid resuscitation corrected sepsis-induced hyponatremia, and multidisciplinary input from ENT, thoracic surgery, and infectious disease specialists ensured comprehensive care, aligning with guidelines emphasizing prompt surgery, antibiotics, and critical care support [8,12,13].

The initial misdiagnosis in an urgent care setting reflects systemic challenges, including high patient volumes, limited laboratory access, and reliance on rapid antigen tests, which can narrow diagnostic focus, as seen in similar cases misdiagnosed as influenza [8,9]. Enhanced clinician training on atypical presentations, improved diagnostic access, and public health strategies like harm reduction for IVDU and better dental care are critical to reducing CNF incidence.

## Conclusions

This case of CNF with DNM in an immunocompromised IVDU patient presenting with intractable hiccups illustrates the perils of misdiagnosis and the critical need for a high index of suspicion in atypical presentations. Hiccups, likely caused by phrenic or vagus nerve irritation or sepsis-related hyponatremia, represent a novel CNF symptom, warranting further investigation. Early LRINEC score application could have expedited diagnosis, highlighting its role as an adjunct to clinical judgment. The successful outcome, driven by timely CT imaging, aggressive surgical debridement, targeted antibiotics, and multidisciplinary care, underscores the efficacy of rapid, coordinated intervention. Clinicians should integrate LRINEC scoring, comprehensive history-taking, and heightened awareness of CNF in immunocompromised patients with nonspecific or worsening symptoms. Public health efforts focusing on infection prevention, substance use reduction, and early recognition of severe infections are essential to improve outcomes in this rapidly progressive, potentially fatal condition.
